# Loss of the bloom syndrome helicase increases DNA ligase 4-independent genome rearrangements and tumorigenesis in aging *Drosophila*

**DOI:** 10.1186/gb-2011-12-12-r121

**Published:** 2011-12-19

**Authors:** Ana Maria Garcia, Robert N Salomon, Alice Witsell, Justine Liepkalns, R Brent Calder, Moonsook Lee, Martha Lundell, Jan Vijg, Mitch McVey

**Affiliations:** 1 Department of Biology, University of Texas at San Antonio, One UTSA Circle, San Antonio, TX 78249, USA; 2 Department of Pathology, Tufts Medical Center, 800 Washington Street, Boston, MA 02111, USA; 3 Department of Biology, Tufts University, 165 Packard Avenue, Medford, MA 02155, USA; 4 Department of Genetics, Albert Einstein College of Medicine, 1301 Morris Park Avenue, Bronx, NY 10461, USA

## Abstract

**Background:**

The BLM DNA helicase plays a vital role in maintaining genome stability. Mutations in *BLM *cause Bloom syndrome, a rare disorder associated with cancer predisposition and premature aging. Humans and mice with *blm *mutations have increased frequencies of spontaneous mutagenesis, but the molecular basis of this increase is not well understood. In addition, the effect of aging on spontaneous mutagenesis in *blm *mutants has not been characterized. To address this, we used a *lacZ *reporter system in wild-type and several mutant strains of *Drosophila melanogaster *to analyze mechanisms of mutagenesis throughout their lifespan.

**Results:**

Our data show that *Drosophila *lacking BLM have an elevated frequency of spontaneous genome rearrangements that increases with age. Although in normal flies most genome rearrangements occur through DNA ligase 4-dependent classical end joining, most rearrangements that accumulate during aging in *blm *mutants do not require DNA ligase 4, suggesting the influence of an alternative end-joining mechanism. Adult *blm *mutants also display reduced lifespan and ligase 4-independent enhanced tumorigenesis in mitotically active tissues.

**Conclusions:**

These results suggest that *Drosophila *BLM suppresses error-prone alternative end-joining repair of DNA double-strand breaks that can result in genome instability and tumor formation during aging. In addition, since loss of BLM significantly affects lifespan and tumorigenesis, the data provide a link between error-prone end joining, genome rearrangements, and tumor formation in a model metazoan.

## Background

Bloom syndrome is a rare, autosomal recessive disorder whose most striking characteristic is a predisposition to all types of cancers (reviewed in [1,2]). It is caused by mutations in the *BLM *gene, a member of the RecQ family of DNA helicases [3]. Cells derived from Bloom syndrome patients and *Blm *hypomorphic mice exhibit greatly elevated genome instability, including a dramatically increased frequency of sister chromatid exchanges [4,5].

Cells with defective BLM also have a heightened mutation frequency that is partially independent of the increase in sister chromatid exchanges [6-9]. The cause of this is poorly understood, but it may be related to the increased number of chromosome aberrations and translocations that are observed in *BLM *mutant cells. Because these types of mutations are hypothesized to be driving forces in the development and progression of cancer in Bloom syndrome patients, understanding their origin is important.

The *mus309 *gene encodes the *Drosophila melanogaster *BLM ortholog DmBlm [10]. Flies lacking DmBlm phenocopy many of the characteristics of human Bloom syndrome, including reduced fertility and increased mitotic sister chromatid exchanges [10-12]. DmBlm is required for accurate homologous recombination (HR) repair of site-specific DNA double-strand breaks (DSBs) and for prevention of mitotic crossovers [13,14]. In its absence, DSB repair frequently proceeds through non-conservative, deletion-prone repair mechanisms. Although initiation of homologous recombination is required for the deletions observed in *blm *mutants [15], the molecular mechanisms responsible for deletion formation remain unknown.

Here, we report that spontaneous mutagenesis at a *lacZ *reporter locus is increased in *Drosophila blm *mutants, similar to what is observed in human Bloom syndrome patients. The increase persists through the adult lifespan and is largely due to elevated genome rearrangements, almost 50% of which involve repetitive genomic regions. Interestingly, although spontaneous rearrangements in wild-type flies depend largely on DNA ligase 4 (Lig4)-dependent classical end joining, rearrangements in *blm *mutants are mostly Lig4-independent. Flies lacking DmBlm, but not Lig4, also have increased tumorigenesis, suggesting that, in the absence of Blm, alternative end joining may be involved in the formation of genome rearrangements that can drive tumorigenesis.

## Results and discussion

### Genome rearrangements involving repeated sequences are common in *blm *mutants

To characterize the consequences of DmBlm deficiency on spontaneous mutagenesis, we crossed a null *mus309 *mutation [12] into a *w^1118 ^*background harboring a *lacZ *reporter construct that allows us to measure somatic mutation frequencies and mutation spectra [16] (Additional file 1). For these studies, we used a *lacZ *reporter integrated at cytological position 87E on chromosome *3*, which exhibits a spontaneous mutation frequency nearly identical to the average obtained using multiple other insertion sites [17]. The *blm *mutant stocks containing the reporter construct were isogenized and homozygous mutants were recovered at days 1, 14, and 28 after adult eclosion. Plasmid rescue was performed and mutation frequencies were calculated on a per locus basis as the number of mutant *lacZ *copies versus the total number of copies rescued from a given amount of DNA (Additional file 2).

**Figure 1:**
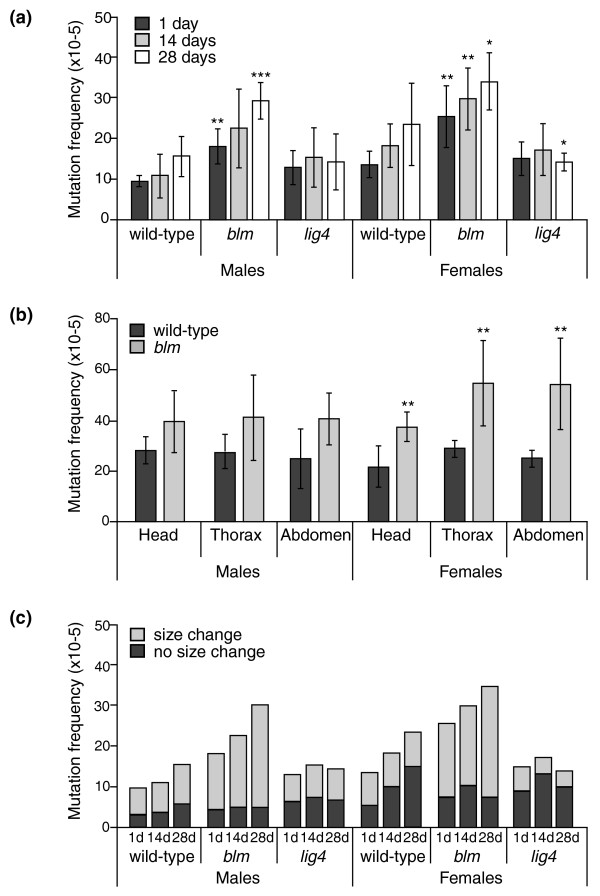
**Somatic mutation frequency and percentage of genome rearrangements increases in *blm *mutants with age**. **(a) **Somatic mutation frequencies grouped by sex and age. Each bar represents five to seven independent measurements. Error bars represent standard deviations. *P < 0.05 compared to wild type, **P < 0.01, ***P < 0.001 (one-way ANOVA). **(b) **Somatic mutation frequencies in various body regions. Samples of head, thorax, and abdominal tissue were collected from 35-day-old male and female flies and somatic mutation frequencies were determined. Each bar represents three to six independent measurements. Error bars represent standard deviations. **P ≤ 0.01 compared to wild type (two-tailed unpaired *t*-test). **(c) **Somatic mutation frequencies that involved either a size change or no size change, grouped by sex and age. Size change mutations correspond to genome rearrangements or *lacZ *internal deletions, while no size change mutations are usually point mutations.

We observed a significantly higher mutation frequency for *blm *mutants relative to wild-type at all ages (Figure 1a; *P *< 0.001 for both sexes, one-way ANOVA). As found previously, mutation frequencies were slightly higher in females than in males [17]. This difference is largely due to an increased frequency of point mutations in females (see below). Since point mutations often result from replication errors, females, which have a larger body size, may have more opportunities to accumulate point mutations.

The increased mutation frequency in *blm *mutants was not specific to a particular region of the fly, but was observed in head, thorax, and abdominal tissues (Figure 1b). Although the differences in tissue-specific mutation frequencies between *blm *mutant and wild-type males were less significant compared to those observed using whole flies, this is likely a consequence of a higher mutation frequency in the wild-type males in this particular experiment (data not shown).

To examine the types of mutations that occurred in *blm *mutants, we performed restriction analysis on a subset of plasmids. Plasmids that show no size change after digestion with the AvaI restriction enzyme generally harbor point mutations, while plasmids showing a size change after digestion involve rearrangements with one breakpoint in the *lacZ *gene and the other elsewhere in the fly genome. We have previously shown that genome rearrangements, including deletions and both intra- and inter-chromosomal translocations, are the predominant type of mutation in flies [17]. Interestingly, the increased mutation accumulation in *blm *mutants appeared to be almost entirely due to an increase in genome rearrangements (Figure 1c). This increase was evident in both males and females (*P *< 0.001, Fisher's exact test).

To further characterize the genome rearrangements recovered from the *blm *mutants, we sequenced the *lacZ *gene in a representative number of mutants and identified the breakpoints. Using BLAST alignment of the recovered sequence against the *Drosophila *genome, we definitively mapped the breakpoints for 11 out of 21 rearrangements (Additional file 3). Three rearrangements were deletions in the *lacZ *transgene, while eight were larger deletions or inversions with the breakpoints outside the reporter gene on the same chromosome. Surprisingly, we were unable to map the breakpoints for 48% of the rearrangements isolated from *blm *mutants because one side of the breakpoint was located in highly repetitive sequences. Six of these sequences corresponded to various transposons, including *accord{}818, stalker*, and *copia*, while four matched repetitive sequences that are highly represented in both euchromatic and heterochromatic regions of the fly genome. Previous studies using the same *lacZ *reporter found a much lower proportion of genome rearrangements involving repetitive sequences (6 out of 55, or 11%, *P *= 0.0003 compared to *blm *mutants, two-tailed Fisher's exact test) [17,18]. Therefore, the increased somatic mutation frequency in *blm *mutants is due, at least in part, to an increased frequency of genome rearrangements involving highly repetitive sequences.

### Differential requirements for rearrangement formation in wild type and *blm *mutants

The simplest model to explain the observed genome rearrangements involves inaccurate repair of two simultaneous DSBs. It is possible that recombination between two breaks could result in deletions and translocations and that DmBlm could suppress these types of rearrangements by preventing homeologous recombination between closely related sequences [19]. However, careful inspection of the junction sequences at the sites of the genome rearrangements isolated from wild type [18] and *blm *mutants did not reveal extensive regions of homology. To determine if classical non-homologous end joining (C-NHEJ) might be required for the formation of these rearrangements, we repeated our analysis in flies lacking DNA ligase 4, which is required for C-NHEJ [20,21]. Surprisingly, we saw no age-dependent increase in mutation frequency in *lig4 *mutants (Figure 1a). In addition, the percentage of genome rearrangements in *lig4 *mutants was significantly less compared to wild-type flies (Figure 1c; *P *≤ 0.01 for both sexes at all ages, two-tailed Fisher's exact test), indicating that Lig4-dependent C-NHEJ is responsible for most of the genome rearrangements that accumulate during normal development and aging.

**Figure 2:**
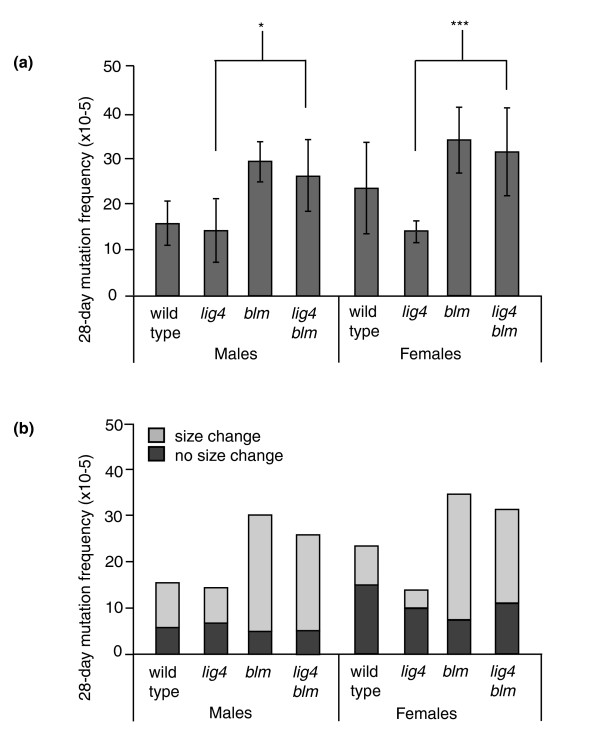
**Increased frequency of genome rearrangements in flies lacking DmBlm is independent of classical non-homologous end joining**. **(a) **Somatic mutation frequencies in 28-day-old flies, grouped by sex. Each bar represents four to six independent measurements. Error bars represent standard deviations. *P < 0.05, ***P < 0.001 compared to wild type (one-way ANOVA). **(b) **Somatic mutation frequencies that involved either a size change or no size change, grouped by sex.

Previous studies from our lab and others have demonstrated that repair of site-specific DSBs in *blm *mutants is frequently accompanied by large deletions [14,15], a feature characteristic of Lig4-independent alternative end joining [22,23]. We therefore wished to determine if the genome rearrangements that occur in *blm *mutants rely on classical or alternative end joining. We constructed *lig4 blm *double mutants containing the *lacZ *reporter construct and measured the mutation frequency and spectra in 28-day-old flies. Spontaneous mutagenesis frequencies for the *lig4 blm *flies were similar to *blm *single mutants, but were significantly different from *lig4 *mutants (Figure 2a; *P *= 0.029 for males, *P *< 0.001 for females, one-way ANOVA). Similarly, the majority of mutations in *lig4 blm *flies were genome rearrangements, paralleling the *blm *mutant phenotype (Figure 2b). In contrast to wild-type flies, therefore, most of the genome rearrangements that occur in flies lacking the DmBlm helicase do not require DNA ligase 4 and are instead generated through an alternative end-joining mechanism.

### Increased tumor formation and reduced lifespan in *blm *mutant flies

**Figure 3:**
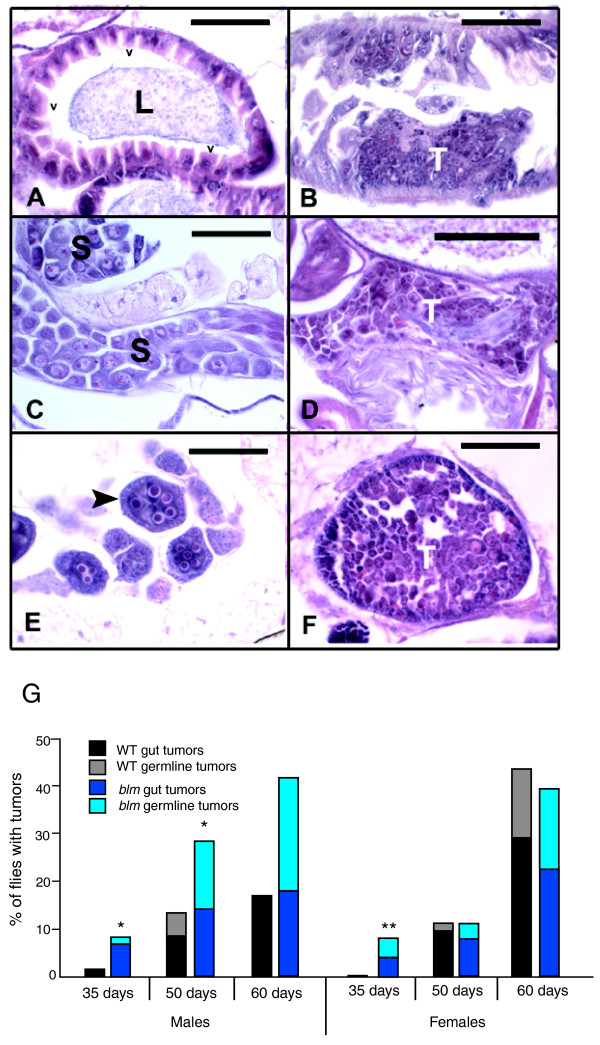
***blm *mutant adults are predisposed to early tumor development**. **(a) **Transverse section through midgut of wild-type fly. Normal, uniform-appearing villi (V) protrude into the gut lumen (L). A cluster of bacteria and yeasts (normal gut flora) is present within the lumen. **(b) **Tumor within the midgut of a *blm *mutant fly. A dense cluster of small tumor cells (T) arises from within the mucosa and forms an irregular mass that protrudes into the gut lumen. **(c) **Normal testis from a wild-type fly. Normal spermatocytes (S) in various stages of development are present. **(d) **Tumor within the testis of a *blm *mutant fly. Large numbers of small tumor cells (T) resembling early germ line precursors have replaced normal spermatocytes within the testis. **(e) **Normal maturing ovarian follicles from a wild-type fly. The largest (arrowhead) contains normal cyst cells surrounded by a single layer of follicular cells. **(f) **Ovarian tumor from a *blm *mutant fly. Large numbers of tumor cells (T) resembling immature germ line precursor cells replace normal cystocytes. All sections were prepared from 35-day-old flies and were stained with hematoxylin and eosin. Each scale bar = 50 microns. **(g) **Histogram showing tumor frequency in 35- to 60-day-old adult flies. *P < 0.05 compared to wild type (WT; two-tailed Fisher's exact test), **P < 0.01.

One hallmark of Bloom syndrome is cancer predisposition at an early age [24]. Recently, several groups have reported that adult flies develop dysplasia and tumors in mitotically active tissues, including intestinal and germline tissues [25,26]. These tumors arise more frequently in older flies [27]. We reasoned that the increased frequency of genome rearrangements observed in the absence of DmBlm might promote tumorigenesis. To test this, we performed histopathological staining on sectioned tissues of wild-type and *blm *mutant flies at 35, 50, and 60 days of age. Representative histological sections from 35-day-old control and age-matched *blm *mutant flies are shown in Figure 3. Normal fly tissues (Figure 3a, c, e) are characterized by a high degree of cytological regularity and orderliness of tissue architecture. In contrast, tumors (Figure 3b, d, f) are composed of densely crowded masses of irregularly arrayed tumor cells. Although no metastases to distant tissues were observed, the gut tumors were frequently observed to partially or fully occlude the intestinal lumen. Germline tumors consisted of masses of proliferating tumor cells that were morphologically similar to stem cells. Like many mammalian tumor cells, individual fly tumor cells were variable in size and shape and showed high nucleus to cytoplasm ratios.

Similar to Bloom syndrome patients, flies lacking DmBlm had an increased frequency of tumor development at early ages. We observed a significant difference in overall tumor formation between 35-day-old wild-type and *blm *mutants (Figure 3g; *P *= 0.05 and *P *= 0.0087 for males and females, respectively). Older males also had increased tumorigenesis relative to wild type (*P *= 0.0047 at 50 days), but tumor frequency was similar in older wild-type and *blm *mutant females. Germline tumors, which are rare in wild-type flies, comprised a significant fraction of tumors in *blm *mutants, particularly in males. This could indicate that certain cell populations within the testes of *blm *males may be more prone to neoplastic transformation. Interestingly, there was no significant difference in tumor frequency between wild type and *lig4 *mutants (Additional file 4), suggesting that increased tumorigenesis is not a result of loss of DSB repair capacity in general. Overall, these results agree with previous findings that tumors accumulate with age in *Drosophila *[27] and indicate that tumorigenesis in flies can be influenced by the status of tumor suppressor genes like *BLM*.

**Figure 4:**
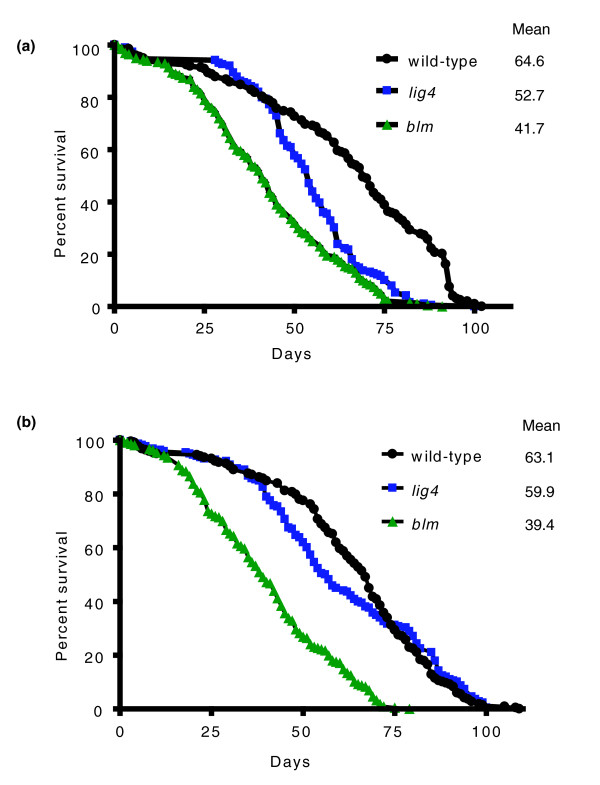
**Loss of Blm decreases mean and maximum lifespan**. **(a) **Kaplan-Meyer survival curves for homozygous wild-type, *lig4*, and *blm *females. Number of individuals: wild type = 218, *lig4 *= 218, *blm *= 216. **(b) **Survival curves for homozygous wild-type, *lig4*, and *blm *males. Number of individuals: wild type = 198, *lig4 *= 189, *blm *= 200. All individuals were unmated. Two independent replicates of these experiments gave similar results.

In *Drosophila*, DSB repair relies more heavily on HR as flies age [28]. Because DmBlm is important for successful completion of HR and plays an important role in the prevention of spontaneous genome rearrangements, we hypothesized that it might also act to promote longevity. To test this, we performed lifespan analysis on isogenic wild-type, *lig4*, and *blm *mutant males and females. Loss of DmBlm significantly shortened mean and maximum lifespan in both sexes (*P *< 0.0001, Wilcoxon rank-sum test; Figure 4). The survivorship plots of the *blm *mutants diverged from the wild-type plots at approximately 20 days, and the mean lifespan of *blm *mutants was reduced more than 35% relative to wild type. In contrast, loss of Lig4 had no significant effect on either the mean or maximum lifespan of female flies (*P *= 0.83). The survivorship plot of the *lig4 *mutant males closely paralleled that of wild-type flies until approximately 45 days, at which point it diverged, intersecting with the *blm *curve near the maximum lifespan of both mutants.

We also quantified tumor formation and lifespan in the double mutants. For both males and females, tumor frequency in *lig4 blm *mutants was similar to levels observed in *blm *single mutants (Additional file 4), matching the spontaneous mutagenesis results (Figure 2). Interestingly, the mean and maximum lifespan of both males and females was further decreased in *lig4 blm *flies relative to *blm *single mutants (Additional file 5). Thus, C-NHEJ repair of DNA DSBs becomes important for organismal lifespan when HR repair is compromised.

## Conclusions

Taken together, our results indicate that genome instability in *Drosophila*, specifically in the form of chromosome rearrangements, is affected to a greater extent by impairment of HR repair than by loss of C-NHEJ. Unlike genome rearrangements that occur during aging in wild-type flies, most rearrangements in *blm *mutants are Lig4-independent. Because *blm *mutants are proficient in the initial resection and strand invasion steps of HR [15], we hypothesize that DSB repair in the absence of DmBlm is more likely to proceed by alternative end-joining mechanisms that involve extensive resection at DNA ends. This leads to an increase in genome rearrangements and may predispose to tumorigenesis and reduced longevity. Interestingly, recent findings demonstrate that alternative end joining also plays a dominant role in translocation formation in mammals [29,30]. In combination with these observations, our study raises the interesting possibility that inaccurate end-joining repair may be a primary cause of pathology in Bloom syndrome and may also be a critical factor in aging.

## Materials and methods

### *Drosophila *stocks and genetics

Flies were raised on standard cornmeal agar medium. All stocks contained a *lacZ *reporter construct integrated at chromosome *3 *position 87E (line *11*) [17]. The *lig4^169a ^*mutation deletes the majority of the *LIG4 *locus, including the promoter [31]. The *mus309^N1 ^*mutation removes the region encoding the amino terminus of DmBlm, including part of the conserved helicase domain [12]. Females from the *lacZ *wild-type and mutant stocks were subjected to at least four backcrosses with *w^1118 ^*males to create isogenic stocks. Heterozygous *lacZ^11 ^mus309^N1^/TM3, lig4^169a^/FM7w; lacZ^11^/TM3*, and *lig4^169a^/FM7w; lacZ^11 ^mus309^N1^/TM3 *stocks were created by standard genetic crosses and mutations were verified by PCR.

### Genomic DNA isolation

For mutation frequency studies, flies were snap frozen at various ages and stored at -80°C. Each sample consisted of 50 pooled male or female flies. For the tissue-specific experiments, each sample consisted of 100 heads, thoraxes, or abdomens that were isolated by cutting snap-frozen flies with a razor blade. Flies were homogenized in 600 μl of lysis buffer (10 mM Tris-HCl, pH 8.0; 10 mM EDTA; 150 mM NaCl) in 2 ml eppendorf tubes using a battery-operated pestle. To the homogenate, 12 μl of Proteinase K (25 mg/ml), 60 μl 10% SDS and 10 μl RNase A (20 μg/ml) were added and samples were incubated at 65°C while rotating during 30 minutes. Genomic DNA was subsequently extracted from these samples using phenol/chloroform as described [16]. DNA yield and quality were estimated by electrophoresis of a small amount of the purified DNA on a 1.0% agarose gel and by spectrophotometric measurement.

### Mutation analysis

The mutation frequency was determined as described in detail elsewhere [16]. Briefly, isolated DNA from 50 flies or 100 body parts was digested for 1 hour at 37°C with HindIII (40 U) in the presence of magnetic beads coated with *lacI-lacZ *fusion protein. The *lacZ *plasmid was then eluted from the beads by incubation with isopropyl β-D-1-thiogalactopyranoside (IPTG), circularized by ligation with T4 DNA ligase (New England Biolabs, Ipswich, MA, USA), precipitated with ethanol and used to electrotransform *Escherichia coli *(Δ*lacZ*, Δ*galE *^-^). Each mutant frequency determination point was based on at least three replicates from the same sample, that is, three 50-fly groups or 100 body parts from the same population, with a minimum of 100, 000 colonies for each rescue. *LacZ *plasmids from mutant colonies were further characterized as described in detail elsewhere [17]. Sequencing reactions of purified mutant plasmids were conducted at the Albert Einstein College of Medicine Genomics core facility. The returned chromatograms were analyzed with Sequencher (Gene Codes, Ann Arbor, MI, USA). Analysis of large rearrangements consisting of non-*lacZ *sequences was carried out using the fly genome database [32]. After alignment with the *D. melanogaster *sequence, the chromosomal origins of the flanking sequences were determined and the orientation and the type of chromosomal rearrangements deduced as described [33].

### Histology

Cohorts of 20 flies were immersed in Telly's fixative (20 parts 70% ethanol, 2 parts 37% formalin, 1 part glacial acetic acid) for a minimum of 48 hours at 4°C prior to processing with a Leica ASP 300 automatic tissue processor using standard techniques. The processed flies were embedded in paraffin blocks, sectioned into ribbons 6 to 7 microns thick, placed onto glass slides, and baked at 65°C for 12 hours to increase tissue adherence prior to staining with hematoxylin and eosin. Digital microphotography was done with an Olympus VANOX-T photomicroscope and a Q Imaging digital camera. All samples were coded and scored without prior knowledge of genotype.

### Lifespan analysis

Homozygous flies collected from isogenized stocks were aged in groups of < 20 flies in vials kept at 25°C and constant humidity on a 12-hour light:12-hour dark cycle. Flies were transferred without anesthesia to new food every 2 to 3 days. For each lifespan experiment, at least 150 flies were monitored and deaths were scored every 1 to 2 days. Lifespan experiments were repeated three times for wild-type and *blm *flies and two times for *lig4 *flies and *lig4 blm *flies.

### Statistical analysis

Somatic mutation frequencies were compared with one-way ANOVA tests followed by Tukey-Kramer multiple comparisons tests for three or more different genotypes and Mann-Whitney tests for comparisons between two genotypes. Gaussian distributions were confirmed using the Kolmogorov and Smirnov method. For tumor frequencies, two-tailed Fisher's exact tests were used to compare between genotypes. Survivorship curves were compared using Wilcoxon matched pairs signed-rank tests. All statistical analysis was conducted using GraphPad Prism software.

## Abbreviations

C-NHEJ: classical non homologous end joining; DmBlm: *Drosophila melanogaster *Blm; DSB: double-strand break; HR: homologous recombination; Lig4: DNA ligase 4.

## Competing interests

The authors declare that they have no competing interests.

## Authors' contributions

AMG designed the study, conducted and analyzed the mutation frequency experiments and wrote the manuscript. MLu designed the study. JV and MM designed the study and wrote the manuscript. MLee conducted and analyzed the mutation frequency experiments. RS carried out the tumorigenesis studies. RBC carried out bioinformatic analysis of the rearrangements. AW and JL carried out the lifespan analysis. All authors read and approved the final manuscript for publication.

## Supplementary Material

Additional file 1 **Methods used to calculate spontaneous mutation frequency and analyze mutations**. Click here for file

Additional file 2 **Information about the numbers of colonies obtained in the mutation frequency experiments**. Click here for file

Additional file 3 **Information about the genome rearrangements isolated in this study**. Click here for file

Additional file 4 **Set of tables showing data related to tumor frequency in wild-type and mutant backgrounds and statistical analysis for tumor frequency in different genetic backgrounds**. Click here for file

Additional file 5 **Lifespan analysis for *lig4 blm *double mutant flies**. Click here for file

## References

[B1] German J Bloom's syndrome. Dermatol Clin 1995 13 7 18 7712653

[B2] Monnat RJ Jr Human RECQ helicases: roles in DNA metabolism, mutagenesis and cancer biology. Semin Cancer Biol 2010 20 329 339 10.1016/j.semcancer.2010.10.002 20934517 PMC3040982

[B3] Ellis NA Groden J Ye TZ Straughen J Lennon DJ Ciocci S Proytcheva M German J The Bloom's syndrome gene product is homologous to RecQ helicases. Cell 1995 83 655 666 10.1016/0092-8674(95)90105-1 7585968

[B4] Chaganti RS Schonberg S German J A manyfold increase in sister chromatid exchanges in Bloom's syndrome lymphocytes. Proc Natl Acad Sci USA 1974 71 4508 4512 10.1073/pnas.71.11.4508 4140506 PMC433916

[B5] Luo G Santoro IM McDaniel LD Nishijima I Mills M Youssoufian H Vogel H Schultz RA Bradley A Cancer predisposition caused by elevated mitotic recombination in Bloom mice. Nat Genet 2000 26 424 429 10.1038/82548 11101838

[B6] Tereshchenko IV Chen Y McDaniel LD Schultz RA Tischfield JA Shao C Small scale genetic alterations contribute to increased mutability at the X-linked Hprt locus in vivo in Blm hypomorphic mice. DNA Repair (Amst) 2010 9 551 557 10.1016/j.dnarep.2010.02.005 20299287

[B7] Warren ST Schultz RA Chang CC Wade MH Trosko JE Elevated spontaneous mutation rate in Bloom syndrome fibroblasts. Proc Natl Acad Sci USA 1981 78 3133 3137 10.1073/pnas.78.5.3133 6942420 PMC319514

[B8] Langlois RG Bigbee WL Jensen RH German J Evidence for increased in vivo mutation and somatic recombination in Bloom's syndrome. Proc Natl Acad Sci USA 1989 86 670 674 10.1073/pnas.86.2.670 2911598 PMC286535

[B9] Tachibana A Tatsumi K Masui T Kato T Large deletions at the HPRT locus associated with the mutator phenotype in a Bloom's syndrome lymphoblastoid cell line. Mol Carcinog 1996 17 41 47 10.1002/(SICI)1098-2744(199609)17:1<41::AID-MC6>3.0.CO;2-N 8876674

[B10] Kusano K Berres ME Engels WR Evolution of the RECQ family of helicases: A *Drosophila *homolog, Dmblm, is similar to the human Bloom syndrome gene. Genetics 1999 151 1027 1039 10049920 10.1093/genetics/151.3.1027 PMC1460517

[B11] Kusano K Johnson-Schlitz DM Engels WR Sterility of *Drosophila *with mutations in the Bloom syndrome gene - complementation by Ku70. Science 2001 291 2600 2602 10.1126/science.291.5513.2600 11283371

[B12] McVey M Andersen SL Broze Y Sekelsky J Multiple functions of *Drosophila *BLM helicase in maintenance of genome stability. Genetics 2007 176 1979 1992 10.1534/genetics.106.070052 17507683 PMC1950607

[B13] Adams MD McVey M Sekelsky JJ *Drosophila *BLM in double-strand break repair by synthesis-dependent strand annealing. Science 2003 299 265 267 10.1126/science.1077198 12522255

[B14] Johnson-Schlitz D Engels WR Template disruptions and failure of double Holliday junction dissolution during double-strand break repair in *Drosophila *BLM mutants. Proc Natl Acad Sci USA 2006 103 16840 16845 10.1073/pnas.0607904103 17075047 PMC1636541

[B15] McVey M Larocque JR Adams MD Sekelsky JJ Formation of deletions during double-strand break repair in *Drosophila *DmBlm mutants occurs after strand invasion. Proc Natl Acad Sci USA 2004 101 15694 15699 10.1073/pnas.0406157101 15501916 PMC524851

[B16] Garcia AM Busuttil RA Rodriguez A Cabrera C Lundell M Dolle ME Vijg J Detection and analysis of somatic mutations at a lacZ reporter locus in higher organisms: application to Mus musculus and *Drosophila *melanogaster. Methods Mol Biol 2007 371 267 287 10.1007/978-1-59745-361-5_20 17634588

[B17] Garcia AM Derventzi A Busuttil R Calder RB Perez E Jr Chadwell L Dolle ME Lundell M Vijg J A model system for analyzing somatic mutations in *Drosophila *melanogaster. Nat Methods 2007 4 401 403 17435764 10.1038/NMETH1027 PMC2723853

[B18] Garcia AM Calder RB Dolle ME Lundell M Kapahi P Vijg J Age- and temperature-dependent somatic mutation accumulation in *Drosophila *melanogaster. PLoS Genet 2010 6 E1000950 10.1371/journal.pgen.1000950 20485564 PMC2869313

[B19] Kappeler M Kranz E Woolcock K Georgiev O Schaffner W *Drosophila *bloom helicase maintains genome integrity by inhibiting recombination between divergent DNA sequences. Nucleic Acids Res 2008 36 6907 6917 10.1093/nar/gkn793 18978019 PMC2588521

[B20] Preston CR Flores CC Engels WR Differential usage of alternative pathways of double-strand break repair in *Drosophila*. Genetics 2006 172 1055 1068 16299390 10.1534/genetics.105.050138 PMC1456205

[B21] Wei DS Rong YS A genetic screen for DNA double-strand break repair mutations in *Drosophila*. Genetics 2007 177 63 77 10.1534/genetics.107.077693 17660539 PMC2013711

[B22] Mladenov E Iliakis G Induction and repair of DNA double strand breaks: the increasing spectrum of non-homologous end joining pathways. Mutat Res 2011 711 61 72 10.1016/j.mrfmmm.2011.02.005 21329706

[B23] Yan CT Boboila C Souza EK Franco S Hickernell TR Murphy M Gumaste S Geyer M Zarrin AA Manis JP Rajewsky K Alt FW IgH class switching and translocations use a robust non-classical end-joining pathway. Nature 2007 449 478 482 10.1038/nature06020 17713479

[B24] German J Bloom syndrome: a mendelian prototype of somatic mutational disease. Medicine 1993 72 393 406 8231788

[B25] Biteau B Hochmuth CE Jasper H JNK activity in somatic stem cells causes loss of tissue homeostasis in the aging *Drosophila *gut. Cell Stem Cell 2008 3 442 455 10.1016/j.stem.2008.07.024 18940735 PMC3225008

[B26] Apidianakis Y Pitsouli C Perrimon N Rahme L Synergy between bacterial infection and genetic predisposition in intestinal dysplasia. Proc Natl Acad Sci USA 2009 106 20883 20888 10.1073/pnas.0911797106 19934041 PMC2791635

[B27] Salomon RN Jackson FR Tumors of testis and midgut in aging flies. Fly (Austin) 2008 2 265 268 19077545 10.4161/fly.7396

[B28] Preston CR Flores C Engels WR Age-dependent usage of double-strand-break repair pathways. Curr Biol 2006 16 2009 2015 10.1016/j.cub.2006.08.058 17055979

[B29] Simsek D Jasin M Alternative end-joining is suppressed by the canonical NHEJ component Xrcc4-ligase IV during chromosomal translocation formation. Nat Struct Mol Biol 2010 17 410 416 10.1038/nsmb.1773 20208544 PMC3893185

[B30] Zhang Y Jasin M An essential role for CtIP in chromosomal translocation formation through an alternative end-joining pathway. Nat Struct Mol Biol 2011 18 80 84 10.1038/nsmb.1940 21131978 PMC3261752

[B31] McVey M Radut D Sekelsky JJ End-joining repair of double-strand breaks in *Drosophila *melanogaster is largely DNA ligase IV independent. Genetics 2004 168 2067 2076 10.1534/genetics.104.033902 15611176 PMC1448732

[B32] FlyBase http://flybase.org/

[B33] Dolle ME Vijg J Genome dynamics in aging mice. Genome Res 2002 12 1732 1738 10.1101/gr.125502 12421760 PMC187544

