# Supplementary material for: Loss of the bloom syndrome helicase increases DNA ligase 4-independent genome rearrangements and tumorigenesis in aging Drosophila
Source: Genome Biol. 2011 Dec 19;12(12):R121. doi: 10.1186/gb-2011-12-12-r121 (PMC3334616; doi:10.1186/gb-2011-12-12-r121)
Supplement: Additional file 1 — Methods used to calculate spontaneous mutation frequency and analyze mutations. [file gb-2011-12-12-r121-S1.PDF]

Supplementary Figure 1

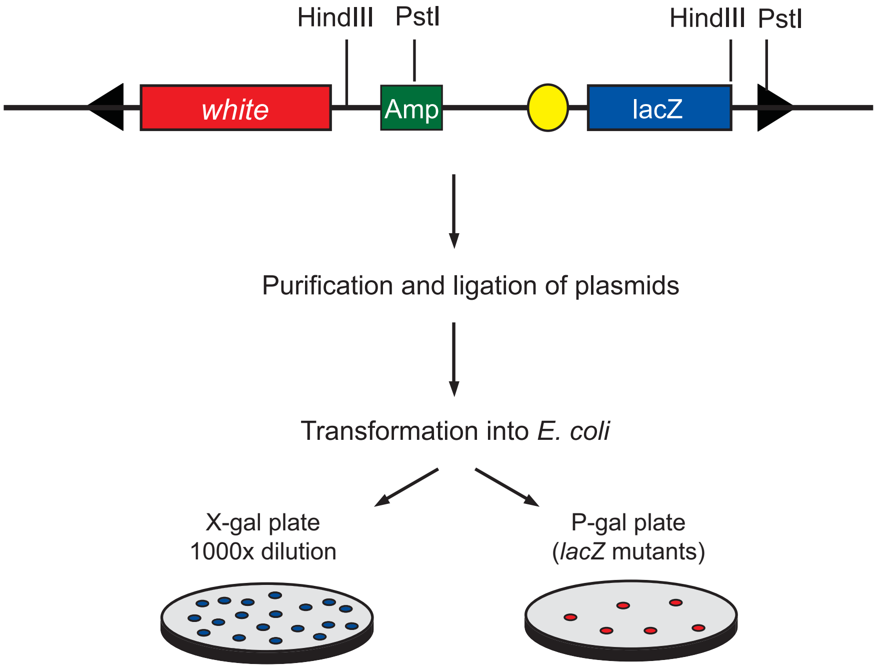

The pUR288 plasmid is contained on the pP[CaSper] *P* element, a single copy of which is integrated into the Drosophila genome at position *87E* on chromosome *3*. The plasmids are purified from fly genomic DNA, self-ligated, and transformed into *Escherichia coli C* (ΔlacZ, galE-) host cells. Individual plasmids are recovered from ampicillin-resistant colonies.

The total number of plasmids rescued is determined by plating a small number of transformants on X-gal medium (titer plate). The rest of the transformation is plated on medium containing the lactose analogue p-gal, which selects cells containing a mutant *lacZ* gene. The mutation frequency is calculated as the ratio of the colonies on the selective plate divided by the colonies on the titer plate (multiplied by the dilution factor).

Black triangles = 5’ and 3’ ends of pP[CaSper], red box = *white* eye color marker, green box = ampicillin resistance gene, yellow circle = origin of replication, blue box = *lacZ* reporter gene.
