# Supplementary material for: Loss of the bloom syndrome helicase increases DNA ligase 4-independent genome rearrangements and tumorigenesis in aging Drosophila
Source: Genome Biol. 2011 Dec 19;12(12):R121. doi: 10.1186/gb-2011-12-12-r121 (PMC3334616; doi:10.1186/gb-2011-12-12-r121)
Supplement: Additional file 2 — Information about the numbers of colonies obtained in the mutation frequency experiments. [file gb-2011-12-12-r121-S2.DOC]

**Supplementary Table 1**. Mutation frequencies for whole flies.

| Genotype | Sex | Age (days) | Trial | # of colonies on X-gal (x10^3^) | # of colonies on P-gal | Mutation frequency (x10^-5^) | Mean +/- S.D. |
| --- | --- | --- | --- | --- | --- | --- | --- |
| wildtype | M | 1 | 1 | 286 | 34 | 11.9 | 9.5 +/- 1.3 |
|  |  |  | 2 | 53 | 5 | 9.4 |
|  |  |  | 3 | N.D. | N.D. | 8.6 |
|  |  |  | 4 | N.D. | N.D. | 8.4 |
|  |  |  | 5 | N.D. | N.D. | 9.9 |
|  |  |  | 6 | N.D. | N.D. | 8.6 |
| wildtype | M | 14 | 1 | 380 | 76 | 20.0 | 11.0 +/- 5.4 |
|  |  |  | 2 | 668 | 47 | 7.0 |
|  |  |  | 3 | 219 | 16 | 7.3 |
|  |  |  | 4 | N.D. | N.D. | 11.5 |
|  |  |  | 5 | N.D. | N.D. | 8.9 |
| wildtype | M | 28 | 1 | 126 | 16 | 12.7 | 15.7 +/- 4.8 |
|  |  |  | 2 | 516 | 94 | 18.2 |
|  |  |  | 3 | 308 | 23 | 7.5 |
|  |  |  | 4 | 365 | 45 | 12.3 |
|  |  |  | 5 | 109 | 21 | 19.3 |
|  |  |  | 6 | 214 | 49 | 22.9 |
|  |  |  | 7 | 760 | 129 | 17.0 |
|  |  |  | 8 | 432 | 69 | 16.0 |
| wildtype | F | 1 | 1 | 410 | 72 | 17.6 | 13.6 +/- 3.3 |
|  |  |  | 2 | 668 | 105 | 15.7 |
|  |  |  | 3 | 354 | 34 | 9.6 |
|  |  |  | 4 | 142 | 19 | 13.4 |
|  |  |  | 5 | N.D. | N.D. | 16.2 |
|  |  |  | 6 | N.D. | N.D. | 13.5 |
|  |  |  | 7 | N.D. | N.D. | 8.9 |
| wildtype | F | 14 | 1 | 359 | 93 | 25.9 | 18.3 +/- 5.3 |
|  |  |  | 2 | 1044 | 95 | 9.1 |
|  |  |  | 3 | 121 | 17 | 14.1 |
|  |  |  | 4 | 285 | 37 | 13.0 |
|  |  |  | 5 | N.D. | N.D. | 18.2 |
|  |  |  | 6 | N.D. | N.D. | 20.1 |
|  |  |  | 7 | N.D. | N.D. | 19.6 |
|  |  |  | 8 | N.D. | N.D. | 20.8 |
|  |  |  | 9 | N.D. | N.D. | 23.5 |
| wildtype | F | 28 | 1 | 264 | 64 | 24.2 | 23.5 +/- 10.0 |
|  |  |  | 2 | 189 | 21 | 11.1 |
|  |  |  | 3 | 277 | 33 | 11.9 |
|  |  |  | 4 | 636 | 157 | 24.7 |
|  |  |  | 5 | 150 | 28 | 18.7 |
| Genotype | Sex | Age (days) | Trial | # of colonies on X-gal (x10^3^) | # of colonies on P-gal | Mutation frequency (x10^-5^) | Mean +/- S.D. |
| wildtype | F | 28 | 6 | 484 | 84 | 17.4 |  |
|  |  |  | 7 | 468 | 185 | 39.5 |
|  |  |  | 8 | 686 | 252 | 36.7 |
|  |  |  | 9 | 550 | 150 | 27.2 |
| *lig4* | M | 1 | 1 | 1052 | 81 | 7.7 | 12.9 +/- 4.6 |
|  |  |  | 2 | 277 | 45 | 16.2 |
|  |  |  | 3 | 115 | 17 | 14.8 |
| *lig4* | M | 14 | 1 | 193 | 42 | 21.8 | 15.4 +/- 7.4 |
|  |  |  | 2 | 270 | 13 | 4.8 |
|  |  |  | 3 | 121 | 20 | 16.5 |
|  |  |  | 4 | 299 | 55 | 18.4 |
| *lig4* | M | 28 | 1 | 377 | 24 | 6.4 | 14.3 +/- 6.9 |
|  |  |  | 2 | 544 | 53 | 9.7 |
|  |  |  | 3 | 147 | 38 | 25.9 |
|  |  |  | 4 | 350 | 55 | 15.7 |
|  |  |  | 5 | 937 | 192 | 20.5 |
|  |  |  | 6 | 1054 | 127 | 12.0 |
|  |  |  | 7 | 943 | 91 | 9.7 |
| *lig4* | F | 1 | 1 | 214 | 40 | 18.7 | 15.1 +/- 4.0 |
|  |  |  | 2 | 1228 | 132 | 10.7 |
|  |  |  | 3 | 497 | 79 | 15.9 |
| *lig4* | F | 14 | 1 | 137 | 29 | 21.2 | 17.2 +/- 6.3 |
|  |  |  | 2 | 108 | 9 | 8.3 |
|  |  |  | 3 | 200 | 44 | 22.0 |
|  |  |  | 4 | 198 | 34 | 17.2 |
| *lig4* | F | 28 | 1 | 254 | 37 | 14.6 | 14.2 +/- 2.2 |
|  |  |  | 2 | 756 | 87 | 11.5 |
|  |  |  | 3 | 285 | 48 | 16.8 |
|  |  |  | 4 | 599 | 91 | 15.2 |
|  |  |  | 5 | 488 | 53 | 10.9 |
|  |  |  | 6 | 745 | 95 | 12.8 |
|  |  |  | 7 | 611 | 94 | 15.4 |
|  |  |  | 8 | 646 | 107 | 16.6 |
| *mus309* | M | 1 | 1 | 760 | 132 | 17.4 | 18.1 +/- 4.2 |
|  |  |  | 2 | 222 | 56 | 25.2 |
|  |  |  | 3 | N.D. | N.D. | 15.2 |
|  |  |  | 4 | N.D. | N.D. | 17.8 |
|  |  |  | 5 | N.D. | N.D. | 19.8 |
|  |  |  | 6 | N.D. | N.D. | 12.9 |
| *mus309* | M | 14 | 1 | 672 | 174 | 25.9 | 22.6 +/- 9.7 |
|  |  |  | 2 | 321 | 80 | 24.9 |
|  |  |  | 3 | 118 | 48 | 40.7 |
| Genotype | Sex | Age (days) | Trial | # of colonies on X-gal (x10^3^) | # of colonies on P-gal | Mutation frequency (x10^-5^) | Mean +/- S.D. |
| *mus309* | M | 14 | 4 | N.D. | N.D. | 13.8 |  |
|  |  |  | 5 | N.D. | N.D. | 15.9 |
|  |  |  | 6 | N.D. | N.D. | 12.7 |
|  |  |  | 7 | N.D. | N.D. | 24.1 |
| *mus309* | M | 28 | 1 | 269 | 68 | 25.3 | 29.3 +/- 4.4 |
|  |  |  | 2 | 230 | 85 | 37.0 |
|  |  |  | 3 | 776 | 224 | 28.9 |
|  |  |  | 4 | 405 | 132 | 32.6 |
|  |  |  | 5 | 269 | 73 | 27.1 |
|  |  |  | 6 | 903 | 249 | 27.6 |
|  |  |  | 7 | 980 | 318 | 32.4 |
|  |  |  | 8 | 1118 | 264 | 23.6 |
| *mus309* | F | 1 | 1 | 159 | 52 | 32.7 | 25.4 +/- 7.5 |
|  |  |  | 2 | 920 | 239 | 26.0 |
|  |  |  | 3 | 262 | 92 | 35.1 |
|  |  |  | 4 | 118 | 28 | 23.7 |
|  |  |  | 5 | N.D. | N.D. | 16.1 |
|  |  |  | 6 | N.D. | N.D. | 18.9 |
| *mus309* | F | 14 | 1 | 122 | 53 | 43.4 | 29.8 +/- 7.5 |
|  |  |  | 2 | 385 | 103 | 26.8 |
|  |  |  | 3 | 214 | 81 | 37.9 |
|  |  |  | 4 | 136 | 42 | 30.9 |
|  |  |  | 5 | N.D. | N.D. | 28.0 |
|  |  |  | 6 | N.D. | N.D. | 18.3 |
|  |  |  | 7 | N.D. | N.D. | 26.8 |
|  |  |  | 8 | N.D. | N.D. | 23.5 |
|  |  |  | 9 | N.D. | N.D. | 32.8 |
| *mus309* | F | 28 | 1 | 150 | 52 | 34.7 | 33.9 +/- 7.1 |
|  |  |  | 2 | 640 | 184 | 28.8 |
|  |  |  | 3 | 900 | 335 | 37.2 |
|  |  |  | 4 | 87 | 35 | 40.2 |
|  |  |  | 5 | 112 | 26 | 23.2 |
|  |  |  | 6 | 338 | 147 | 43.5 |
|  |  |  | 7 | 447 | 134 | 30.0 |
| *lig4 mus309* | M | 28 | 1 | 721 | 175 | 24.3 | 26.1 +/- 7.9 |
|  |  |  | 2 | 419 | 155 | 37.0 |
|  |  |  | 3 | 451 | 113 | 25.1 |
|  |  |  | 4 | 599 | 108 | 18.0 |
| *lig4 mus309* | F | 28 | 1 | 504 | 126 | 25.0 | 31.4 +/- 9.6 |
|  |  |  | 2 | 346 | 162 | 46.8 |
| Genotype | Sex | Age (days) | Trial | # of colonies on X-gal (x10^3^) | # of colonies on P-gal | Mutation frequency (x10^-5^) | Mean +/- S.D. |
| *lig4 mus309* | F | 28 | 3 | 475 | 152 | 32.0 |  |
|  |  |  | 4 | 396 | 106 | 26.8 |
|  |  |  | 5 | 294 | 110 | 37.4 |
|  |  |  | 6 | 335 | 68 | 20.3 |

N.D. = no data (actual colony counts could not be found)

**Supplementary Table 2**. Mutation frequencies for different body regions.

| Genotype | Sex | Body Part | Trial | # of colonies on X-gal (x10^3^) | # of colonies on P-gal | Mutation frequency (x10^-5^) | Mean +/- S.D. |
| --- | --- | --- | --- | --- | --- | --- | --- |
| wildtype | M | Head | 1 | 239 | 86 | 36.0 | 28.0 +/- 5.4 |
|  |  |  | 2 | 224 | 70 | 26.8 |
|  |  |  | 3 | 177 | 44 | 24.9 |
|  |  |  | 4 | 229 | 56 | 24.5 |
| wildtype | M | Thorax | 1 | 422 | 100 | 23.7 | 27.2 +/- 6.5 |
|  |  |  | 2 | 263 | 74 | 28.1 |
|  |  |  | 3 | 412 | 108 | 26.2 |
|  |  |  | 4 | 262 | 101 | 38.5 |
|  |  |  | 5 | 460 | 88 | 19.1 |
|  |  |  | 6 | 190 | 52 | 27.4 |
| wildtype | M | Abdomen | 1 | 471 | 88 | 18.7 | 24.8 +/- 11.8 |
|  |  |  | 2 | 322 | 84 | 26.1 |
|  |  |  | 3 | 150 | 61 | 40.7 |
|  |  |  | 4 | 234 | 32 | 13.7 |
| *blm* | M | Head | 1 | 409 | 101 | 24.7 | 39.4 +/- 11.9 |
|  |  |  | 2 | 279 | 98 | 35.1 |
|  |  |  | 3 | 339 | 171 | 50.4 |
|  |  |  | 4 | 236 | 112 | 47.5 |
| *blm* | M | Thorax | 1 | 590 | 175 | 29.7 | 41.1 +/- 16.9 |
|  |  |  | 2 | 475 | 139 | 29.3 |
|  |  |  | 3 | 389 | 157 | 40.4 |
|  |  |  | 4 | 236 | 154 | 65.3 |
| *blm* | M | Abdomen | 1 | 253 | 78 | 30.8 | 40.5 +/- 10.2 |
|  |  |  | 2 | 428 | 169 | 39.5 |
|  |  |  | 3 | 371 | 190 | 51.2 |
| wildtype | F | Head | 1 | 268 | 93 | 34.7 | 21.5 +/- 8.2 |
|  |  |  | 2 | 442 | 77 | 17.4 |
|  |  |  | 3 | 315 | 56 | 17.8 |
|  |  |  | 4 | 223 | 31 | 13.9 |
|  |  |  | 5 | 412 | 97 | 23.5 |
| wildtype | F | Thorax | 1 | 628 | 174 | 27.7 | 28.9 +/- 3.0 |
|  |  |  | 2 | 612 | 183 | 29.9 |
|  |  |  | 3 | 313 | 96 | 30.7 |
|  |  |  | 4 | 464 | 113 | 24.4 |
|  |  |  | 5 | 520 | 166 | 31.9 |
| wildtype | F | Abdomen | 1 | 468 | 129 | 27.6 | 25.0 +/- 3.2 |
|  |  |  | 2 | 173 | 24 | 26.0 |
|  |  |  | 3 | 943 | 196 | 20.8 |
|  |  |  | 4 | 383 | 108 | 28.2 |
|  |  |  | 5 | 415 | 94 | 22.7 |
| Genotype | Sex | Body Part | Trial | # of colonies on X-gal (x10^3^) | # of colonies on P-gal | Mutation frequency (x10^-5^) | Mean +/- S.D. |
| *blm* | F | Head | 1 | 145 | 57 | 39.3 | 37.2 +/- 5.7 |
|  |  |  | 2 | 343 | 103 | 30.0 |
|  |  |  | 3 | 202 | 73 | 36.1 |
|  |  |  | 4 | 397 | 184 | 46.3 |
|  |  |  | 5 | 427 | 140 | 32.8 |
|  |  |  | 6 | 252 | 97 | 38.5 |
| *blm* | F | Thorax | 1 | 250 | 93 | 37.2 | 54.5 +/- 16.9 |
|  |  |  | 2 | 523 | 212 | 40.5 |
|  |  |  | 3 | 143 | 65 | 45.5 |
|  |  |  | 4 | 234 | 126 | 53.8 |
|  |  |  | 5 | 294 | 213 | 72.4 |
|  |  |  | 6 | 168 | 139 | 77.4 |
| *blm* | F | Abdomen | 1 | 273 | 146 | 53.5 | 54.0 +/- 17.8 |
|  |  |  | 2 | 475 | 186 | 39.2 |
|  |  |  | 3 | 63 | 21 | 33.3 |
|  |  |  | 4 | 550 | 262 | 47.6 |
|  |  |  | 5 | 288 | 211 | 73.3 |
|  |  |  | 6 | 353 | 272 | 77.1 |
