# Supplementary material for: Loss of the bloom syndrome helicase increases DNA ligase 4-independent genome rearrangements and tumorigenesis in aging Drosophila
Source: Genome Biol. 2011 Dec 19;12(12):R121. doi: 10.1186/gb-2011-12-12-r121 (PMC3334616; doi:10.1186/gb-2011-12-12-r121)
Supplement: Additional file 3 — Information about the genome rearrangements isolated in this study. [file gb-2011-12-12-r121-S3.DOC]

Supplementary Table 3. Sequenced size-change mutants recovered from *blm* mutants

| Sample ID | Type of event | 5' sequence at breakpoint | 3' sequence at breakpoint | Location 3' breakpoint^1^ | Position 3' breakpoint^2^ | Position 5' breakpoint^3^ | Orientation 5' breakpoint^4^ | Distance^5^ |
| --- | --- | --- | --- | --- | --- | --- | --- | --- |
| 1 | internal rearrangement | ATCCTTTGCGAATACGCCCA | GCAAGAGCAACTCGGTCGCC | reporter construct | 1837 | 3799 | - | 1961 |
| 16 | internal deletion | GGCTGAAGTTCAGATGTGCG | ATGAAGCCAATATTGAAACC | reporter construct | 960 | 1450 | - | 490 |
| 20 | internal deletion | TGAGCGGCATTTTCCGTGAC | TCTGCTGCCGCTGCGTTTTG | reporter construct | 851 | 3303 | - | 2452 |
| 3 | deletion | GCCCACGCGATGGGTAACAG | CTGCGCTGGCCGCCCGCACC | 3R | 1852 | 910721 | P | 5453 |
| 6 | deletion | GAAAACGGTCTGCGCTGCGG | AAACTCAGCGTCGGAAGCTG | 3R | 3037 | 906513 | P | 8476 |
| 10 | deletion | ACCAGCGAATACCTGTTCCG | CCGTTCAGTTCGGCGGCCAC | 3R | 2152 | 910175 | P | 5699 |
| 11 | deletion | GCTATGACCATGATTACGGA | GAACATAACAAGCCTGCAGC | 3R | 232 | 809441 | P | 108353 |
| 14 | deletion | CACGACGCGCTGTATCGCTG | TTGGGGAGTTGGAGTTGGGG | 3R | 1639 | 905965 | P | 10422 |
| 17 | inversion | TATGGCAGGGTGAAACGCAG | TAGCAGCAGCAGGGTCTTTT | 3L | 1016 | 5820974 | P | 6737984 |
| 21 | inversion | GGAAGCAAAACACCAGCAGC | GGGTCAATGTGCAACTGGTG | 3R | 2091 | 1006319 | D | 101530 |
| 23 | Inversion | - | - | 3R | - | ~11413000 | D | ~10499310 |
| 4 | unresolved chromosomal rearrangement | - | - | undefined c’some | - | *P* element | - | - |
| 7 | unresolved chromosomal rearrangement | ATTTGCCCGATGTACGCGCG | AGTTACGGGTGCCTCCTCCT | undefined c’some | 1732 | *accord{}818* transposon^7^ | - | - |
| 8 | unresolved chromosomal rearrangement | ACACCGCATCCGGCGCGGAT | AATAACCGTGGAGGTAGGCG | multiple^6^ | 2863 | *stalker* transposon^7^ | - | - |
| 9 | unresolved chromosomal rearrangement | ATTTGCCCGATGTACGCGCG | AGTTACGGGTGCCTCCGTTG | multiple^6^ | 1752 | *accord{}818* transposon^7^ | - | - |
| 13 | unresolved chromosomal rearrangement | TACAGGAAGGCCAGACGCGA | TGTTGGAATATACTATTCAA | multiple^6^ | 638 | *copia* retro-transposon^7^ | - | - |
| 15 | unresolved chromosomal rearrangement | GGCGGTGAAATTATCGATGA | AGTTACCATGCCCAGCATTA | undefined c’some | 1060 | *accord{}818* transposon^7^ | - | - |
| 18 | unresolved chromosomal rearrangement | CGTTTGTTCCCACGGAGAAT | TTGACCCAATGGCCTTCTCA | multiple^6^ | 572 | *Dm88* (*S*-element)^7^ | - | - |
| 19 | unresolved chromosomal rearrangement | - | - | multiple^6^ | - | *accord{}818* transposon^7^ | - | - |
| 22 | unresolved intra-chromosomal event | - | - | 3R | - | ~908500 | - | ~5000 |
| 24 | unresolved chromosomal rearrangement | - | - | multiple^6^ | - | - | - | - |

^1^ Chromosomal location of the breakpoint or position of the event when inside the lacZ reporter

^2^ Position of the breakpoint(s) in the lacZ gene (see Garcia, et al. Nat Methods. 2007 for the lacZ sequence)

^3^ Position of the breakpoint in the Drosophila genome as inferred from the 5' genomic sequence captured in the lacZ reporter (see Garcia, et al. Nat. Meth., 2007)

^4^ Inferred orientation of the mapped breakpoint sequence. Distal (D, toward the telomere) or proximal (P, toward the centromere)

^5^ Distance covered by the event, in base pairs

^6^ Multiple high-confidence matches to different genomic scaffolds

^7^ Multiple high-confidence matches on the same chromosome.
