# Supplementary material for: Loss of the bloom syndrome helicase increases DNA ligase 4-independent genome rearrangements and tumorigenesis in aging Drosophila
Source: Genome Biol. 2011 Dec 19;12(12):R121. doi: 10.1186/gb-2011-12-12-r121 (PMC3334616; doi:10.1186/gb-2011-12-12-r121)
Supplement: Additional file 4 — Set of tables showing data related to tumor frequency in wild-type and mutant backgrounds and statistical analysis for tumor frequency in different genetic backgrounds. [file gb-2011-12-12-r121-S4.DOC]

Supplementary Table 4. Tumor incidence is affected by Blm status and adult age

| Age | Sex | Genotype | Number of flies examined | Percent of flies with gut tumors | Percent of flies with  germline tumors | Overall percent of flies with tumors |
| --- | --- | --- | --- | --- | --- | --- |
| 35 days | Male | wild-type | 80 | 1.3 | 0 | 1.3 |
|  |  | *lig4* | 60 | 5.0 | 0 | 5.0 |
|  |  | *mus309* | 74 | 6.8 | 1.4 | 8.1 |
|  |  | *lig4; mus309* | 40 | 17.5 | 0 | 17.5 |
|  | Female | wild-type | 82 | 0 | 0 | 0 |
|  |  | *lig4* | 60 | 0 | 0 | 0 |
|  |  | *mus309* | 100 | 4 | 4 | 8 |
|  |  | *lig4; mus309* | 40 | 7.5 | 2.5 | 10 |
| 50 days | Male | wild-type | 62 | 8.1 | 4.8 | 12.9 |
|  |  | *lig4* | 20 | 0 | 0 | 0 |
|  |  | *mus309* | 64 | 14.1 | 14.1 | 28.1 |
|  |  | *lig4; mus309* | 40 | 27.5 | 12.5 | 40.0 |
|  | Female | wild-type | 64 | 9.4 | 1.6 | 10.9 |
|  |  | *lig4* | 20 | 0 | 0 | 0 |
|  |  | *mus309* | 63 | 7.9 | 3.2 | 11.1 |
|  |  | *lig4; mus309* | 40 | 12.5 | 5.0 | 17.5 |
| 60 days | Male | wild-type | 12 | 16.7 | 0 | 16.7 |
|  |  | *mus309* | 17 | 17.6 | 23.5 | 41.2 |
|  | Female | wild-type | 14 | 28.6 | 14.3 | 42.9 |
|  |  | *mus309* | 18 | 22.2 | 16.7 | 38.9 |

Supplementary Table 5. Statistical comparisons of tumor frequencies, 35-day old females

| Genotype | WT | *lig4* | *mus309* | *lig4; blm* |
| --- | --- | --- | --- | --- |
| WT | X | N.A. | .009 | .01 |
| *lig4* |  | X | .026 | .023 |
| *blm* |  |  | X | .742 |
| *lig4; blm* |  |  |  | X |

*P* values were calculated using overall frequencies of tumors (both gut and germline) and two-tailed Fisher’s exact tests between indicated genotypes. N.A. indicates the calculation could not be done. Yellow shading indicates a *P* value <0.05.

Supplementary Table 6. Statistical comparisons of tumor frequencies, 35-day old males

| Genotype | WT | *lig4* | *blm* | *lig4; blm* |
| --- | --- | --- | --- | --- |
| WT | X | .314 | .05 | .002 |
| *lig4* |  | X | .731 | .084 |
| *blm* |  |  | X | .215 |
| *lig4; blm* |  |  |  | X |

*P* values were calculated using overall frequencies of tumors (both gut and germline) and two-tailed Fisher’s exact tests between indicated genotypes. Yellow shading indicates a *P* value <0.05.

Supplementary Table 7. Statistical comparisons of tumor frequencies, 50-day old females

| Genotype | WT | *lig4* | *blm* | *lig4; blm* |
| --- | --- | --- | --- | --- |
| WT | X | .189 | 1 | .384 |
| *lig4* |  | X | .189 | .084 |
| *blm* |  |  | X | .388 |
| *lig4; blm* |  |  |  | X |

*P* values were calculated using overall frequencies of tumors (both gut and germline) and two-tailed Fisher’s exact tests between indicated genotypes.

Supplementary Table 8. Statistical comparisons of tumor frequencies, 50-day old males

| Genotype | WT | *lig4* | *blm* | *lig4; blm* |
| --- | --- | --- | --- | --- |
| WT | X | .189 | .047 | .004 |
| *lig4* |  | X | .005 | .0005 |
| *blm* |  |  | X | .283 |
| *lig4; blm* |  |  |  | X |

*P* values were calculated using overall frequencies of tumors (both gut and germline) and two-tailed Fisher’s exact tests between indicated genotypes. Yellow shading indicates a *P* value <0.05.
