# Supplementary material for: Loss of the bloom syndrome helicase increases DNA ligase 4-independent genome rearrangements and tumorigenesis in aging Drosophila
Source: Genome Biol. 2011 Dec 19;12(12):R121. doi: 10.1186/gb-2011-12-12-r121 (PMC3334616; doi:10.1186/gb-2011-12-12-r121)
Supplement: Additional file 5 — Lifespan analysis for lig4 blm double mutant flies. [file gb-2011-12-12-r121-S5.PDF]

Supplementary Figure 2

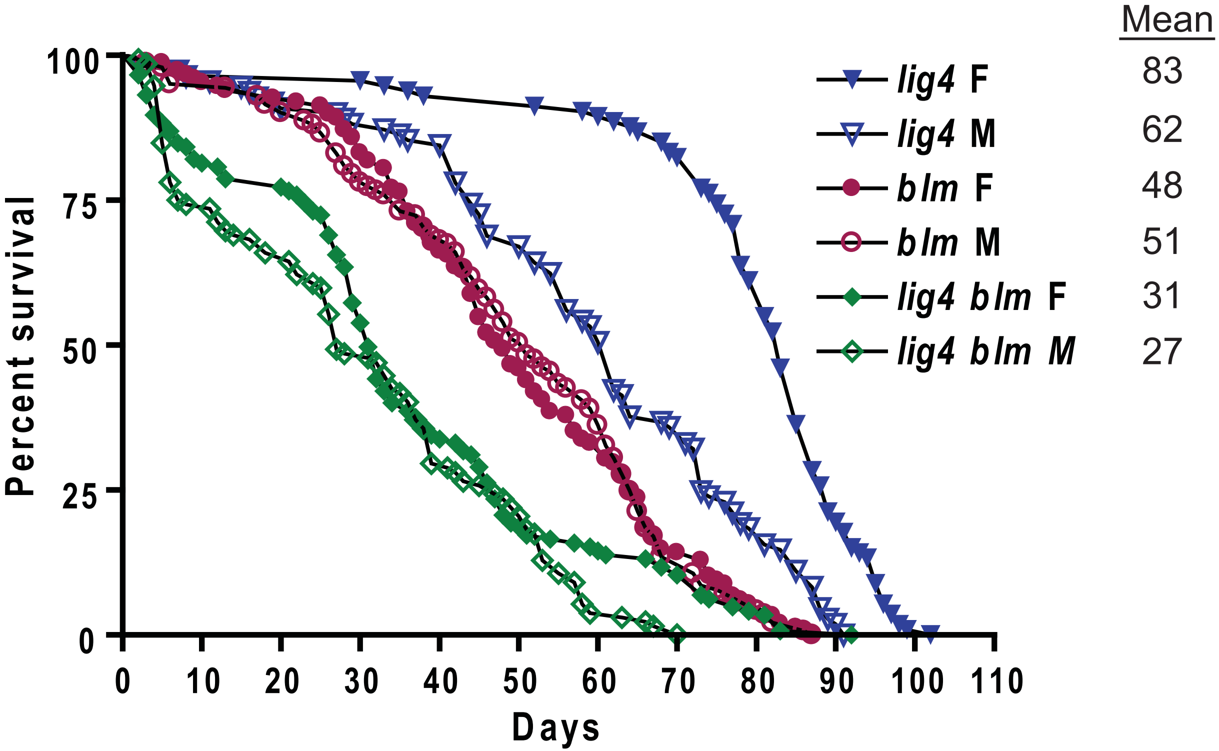

Loss of both DNA ligase 4 and DmBlm further reduces lifespan.
Survival curves for homozygous *lig4*, *blm*, and *lig4;blm* flies.
Number of female individuals: *lig4*=113, *blm*=148, *lig4;blm*=145.
Number of male individuals: *lig4*=109, *blm*=141, *lig4;blm*=132. All individuals were unmated.
